# Stiffness Considerations for a MEMS-Based Weighing Cell

**DOI:** 10.3390/s23063342

**Published:** 2023-03-22

**Authors:** Karin Wedrich, Valeriya Cherkasova, Vivien Platl, Thomas Fröhlich, Steffen Strehle

**Affiliations:** 1 Microsystems Technology Group, Institute of Micro- and Nanotechnologies MacroNano^®^, Technische Universität Ilmenau, Max-Planck-Ring 12, 98693 Ilmenau, Germany; karin.wedrich@tu-ilmenau.de; 2 Force Measurement and Weighing Technology Group, Institute of Process Measurement and Sensor Technology, Technische Universität Ilmenau, Gustav-Kirchhoff-Str. 1, 98693 Ilmenau, Germany; 3 Mechanics of Compliant Systems Group, Technische Universität Ilmenau, Max-Planck-Ring 12, 98693 Ilmenau, Germany

**Keywords:** microsensor, balance, force, stiffness, force compensation

## Abstract

In this paper, a miniaturized weighing cell that is based on a micro-electro-mechanical-system (MEMS) is discussed. The MEMS-based weighing cell is inspired by macroscopic electromagnetic force compensation (EMFC) weighing cells and one of the crucial system parameters, the stiffness, is analyzed. The system stiffness in the direction of motion is first analytically evaluated using a rigid body approach and then also numerically modeled using the finite element method for comparison purposes. First prototypes of MEMS-based weighing cells were successfully microfabricated and the occurring fabrication-based system characteristics were considered in the overall system evaluation. The stiffness of the MEMS-based weighing cells was experimentally determined by using a static approach based on force-displacement measurements. Considering the geometry parameters of the microfabricated weighing cells, the measured stiffness values fit to the calculated stiffness values with a deviation from −6.7 to 3.8% depending on the microsystem under test. Based on our results, we demonstrate that MEMS-based weighing cells can be successfully fabricated with the proposed process and in principle be used for high-precision force measurements in the future. Nevertheless, improved system designs and read-out strategies are still required.

## 1. Introduction

The measurement of small forces is indispensable for various applications in different fields that range from nanoscale metrology to microbiology and quantum physics [1,2,3]. The most frequently used principle for the measurement of small forces is a microcantilever. Such cantilevers are commonly used, for instance, in atomic force microscopy to reconstruct a specimen surface with respect to interaction forces between the surface and a nanoscale scanning probe [3,4,5]. The overall basic working principle of a deflection of a beam due to an input force, combined with relatively low fabrication costs makes this force sensing approach attractive. Optical and piezoresistive concepts are frequently applied, for the readout of the beam deflection.

However, there are some aspects of a microcantilever that must be considered critically. The knowledge of the cantilever stiffness is essential for different reasons. Firstly, the stiffness has a linear influence on the measurement accuracy, which is also true for absolute force measurements, and therefore needs to be determined precisely. Furthermore, the stiffness must fit the input forces to be measured so that the beam deflects adequately. If the deflections are too large, resulting from insufficient stiffness, the measurement accuracy can be affected by occurring nonlinearities or even result in cantilever breakage. Therefore, knowledge of the cantilever stiffness is essential. Nevertheless, the stiffness variation of microcantilevers due to the manufacturing process is typically in the range of only 30%.

To precisely measure macroscopic forces in a wide range, the principle of an electromagnetic force compensation (EMFC) balance is widely used [6,7,8]. Additionally, the principle of force compensation enables a constant accuracy over the entire measurement range. First microscale systems based on the intriguing force compensation principle are shown in [9,10,11,12,13,14,15,16]. Nevertheless, to push the limits of the current approaches requires besides the general system design and microfabrication protocols, a thorough mechanical analysis that considers at least the impact of the real system geometries. Influences of the fabrication process on the stiffness as well as the investigations of the effects of shear forces on the overall mechanisms have not yet been considered.

Inspired by the macroscopic force-compensation weighing cells, a microscopic force compensation mechanism is presented and its stiffness is characterized in correlation with microfabrication. The focus is not only on the system stiffness in the direction of motion but also in out-of-plane direction, which has not been investigated in other microscopic force compensation mechanisms so far. Furthermore, various aspects such as the analytical and numerical stiffness calculation, the microfabrication process, the influence of real system geometries on the stiffness and first stiffness measurements based on a force-displacement measurement are discussed. The comparison of the measured stiffness values to the calculated values shows an agreement with a deviation to each other from −6.8 to 3.8% depending on the investigated system and adapted geometry parameters.

## 2. Material and Methods

**Figure 1:**
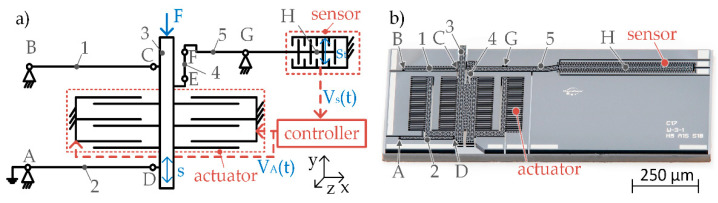
(**a**) Schematic illustration of the working principle of the MEMS-based weighing balance including the mechanical parts (black), the electrical parts and the electrical periphery (red). (1) upper lever, (2) lower lever, (3) shuttle, (4) coupling element, (5) transmission lever, *V_s_* voltage of the sensor signal, *V_A_* Voltage to actuate the shuttle in balance; (**b**) Photo-stacked overview image of the microfabricated MEMS-based weighing cell.

The basic mechanism studied in this work is inspired by macroscopic EMFC balances, as described in detail in [17]. The implemented microscale mechanisms that enable an in-plane movement consist of a guiding and a transmission mechanism as shown in Figure 1. The guiding mechanism allows a linear movement of the shuttle (3) and, in dependence of its stiffness, transmits the input force to a deflection *s*, which is limited to 5 µm by inherent mechanical stops. The guiding mechanism resembles a parallelogram linkage with an upper (1) and a lower lever (2) and four flexure hinges (A–D). The deflection *s* is transferred to a transmission lever with a coupling element (4) and two flexure hinges (E, F), and amplified to an output deflection $s_{t}$ (see hinge H in Figure 1). This mechanism is mainly used to increase the resolution of the position read-out. The MEMS-based weighing cell includes a microfabricated mechanical and an electrical system. A MEMS-based weighing balance further includes peripherals such as housing, other electronics, and a controller.

To detect the deflected shuttle position, a differential electrostatic comb sensor is used because of its very high resolution, which transduces the position into an electrical voltage signal. The controller is used to determine the required microactuator voltage, to move the shuttle back into its zero position. The utilized differential arrangement enables the operation of the sensor independently of environmental influences like temperature or air humidity. The microactuator is an asymmetric electrostatic plate actuator with several fingers *n* in in-plane direction, which can generate comparatively high forces. The force of the actuator $F_{actuator}$ can be calculated according to Equation (1):

(1) $$F_{actuator}=\frac{1}{2}V^{2}\frac{dC\left(y\right)}{dy}=\frac{1}{2}\varepsilon_{0}\varepsilon_{r}AV^{2}\left(\frac{n}{{\left(d_{10}-s\right)}^{2}}-\frac{n}{{\left(d_{20}+s\right)}^{2}}\right)$$

Here $\varepsilon_{0}$ is the vacuum permittivity, $\varepsilon_{r}$ is the relative permittivity, *A* the overlapping area of the actuator plates, $d_{10}$ the initial distance of the plate couples contributing to the force and $d_{20}$ the initial distance of the plates working against the actuator force. An additional advantage of an asymmetric plate actuator is that the resolution increases as the distance between the capacitor plates decreases, as shown in Equation (1). Here the distance between the plates is a quadratic and reciprocal term in the force equation.

The resolution of the weighing cell, as per Equation (2), depends on the stiffness of the system *c*, the resolution of the position sensor $\Delta s_{t}$ and the transmission rate $t_{r}.$

(2) $$\Delta F=c\Delta s_{t}\frac{1}{t_{r}}\;\mathrm{with}\;t_{r}=\frac{l_{GH}}{l_{FG}}$$

### 2.1. Stiffness of the MEMS-Based Weighing Cell

The stiffness of the MEMS-based weighing cell is an important parameter for the achievable resolution of the system as shown in Equation (2). Therefore, a stiffness evaluation of the mechanism is required. It is first determined analytically using a rigid body approach and then also modeled numerically using the finite element method. The comparison of both approaches and the results are presented in the result Section 3.

#### 2.1.1. Analytical Stiffness Calculation

**Figure 2:**
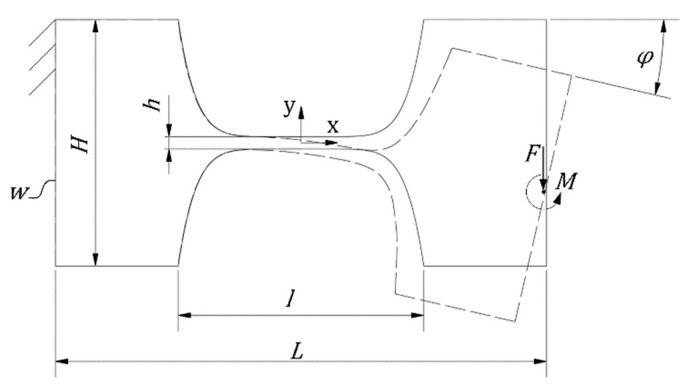
Schematic illustration of a single flexure hinge with indicated elastic deformation as used for the calculation and geometric parameters: maximum height of the hinge *H*, minimal hinge size *h*, width (out-of-plane thickness) of the hinge *w*, length of the hinge *l*, bending angle *φ*, force *F* and momentum *M*.

The calculation of the stiffness of the single flexure hinges of the MEMS-based weighing cell, see in Figure 1 hinges A–G, is performed using the so-called detasFLEX software, which was specifically developed for the analysis of flexure hinges with various geometries at Technische Universität Ilmenau as described in further detail in Reference [18]. The software performs the calculation of elasto-kinematic flexure hinge properties by numerically solving a system of four non-linear differential equations describing a slender beam element with an axially non-extensible neutral axis. These equations are based on the non-linear analytical approach for large deflections of rod-like structures, assuming that the Bernoulli hypothesis, the Saint-Vernant-principle and Hooke’s law apply [18]. Flexure hinges, as illustrated in Figure 2, were investigated with the design parameters (Table 1, Section 3). By applying the input parameter φ in ° present at the end of the flexure hinges, the corresponding moment in Nµm the hinge stiffness in Nµm/° was derived. Regarding the following analytical calculation, the hinge stiffness of a single hinge is converted from Nµm/° to Nµm/rad.

For the analytical stiffness calculation of the entire mechanism, a rigid body model is used to describe the MEMS-based weighing cell like Reference [6]. The thin flexure hinges are assumed to possess perfect rotational joints with a fixed rotational axis without friction and are represent therefore torsional springs with a constant spring stiffness $c_{t}$. The levers between the flexure hinges as well as the shuttle are assumed to be rigid bodies.

The basis for the analytical description is the Lagrange equation, which is the equation of motion of a mechanical system (Equation (2)):

(3) $$\frac{d}{dt}\frac{\partial L}{\partial {\dot{q}}_{i}}-\frac{\partial L}{\partial q_{i}}=Q_{i}\;\;\;\;\;\;\;\;\;\;\;\;\;L=T-U;i=1,\;2,\ldots ,\;f$$

**Figure 3:**
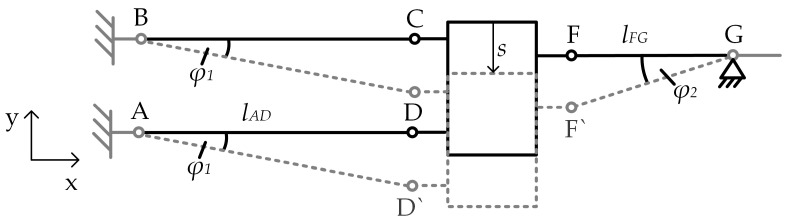
Simplified schematic of the MEMS-based weighing cell that was used for the analytical approach. Black: unloaded state, dashed grey: loaded/deflected state.

L represents the Lagrangian, T the kinetic energy, U the potential energy, q the generalized coordinates, Q the generalized forces, i the number of independent system variables and f the degree of freedom of the mechanical system. Due to the constraint of the guiding mechanism, which only allows for motion in *y*-direction, the degree of freedom of the system is f=1. Therefore, the angle *φ*_2_ of the transmission lever (Figure 3) is sufficient as the single system variable. These assumptions lead to a conservative system, except for the force applied to the shuttle (F):

(4) $$\frac{d}{dt}\frac{\partial L}{\partial {\dot{\varphi }}_{2}}-\frac{\partial L}{\partial \varphi_{2}}=F$$

For a quasi-static description of the system, the velocity of the angle and therefore the kinetic energy T is zero and Equation (4) can be thus reduced to:

(5) $$\frac{\partial U}{\partial \varphi_{2}}=F$$

Furthermore, the deflection of the flexure hinge E is negligible compared to the movement range of the entire mechanism. A kinematically equivalent system is derived and a schematic of the mechanism without the flexure hinge E is shown in Figure 3. The reduced mechanism consists of the parallelogram linkage and the transmission mechanism, which are connected through the shuttle.

The elastic potential energy of torsion springs can generally be calculated according to Equation (6) from which the elastic potential energy of the hinges in the entire mechanism is obtained according to Equation (7):

(6) $$U_{el}=\frac{1}{2}c_{t}\varphi^{2}$$

(7) $$U_{el}=\frac{1}{2}({c_{t}}_{A}+{c_{t}}_{B}+{c_{t}}_{C}+{c_{t}}_{D})\varphi_{1}{}^{2}+\frac{1}{2}({c_{t}}_{F}+{c_{t}}_{G})\varphi_{2}{}^{2}$$

${c_{t}}_{A},\;{c_{t}}_{B},\;\ldots ,\;{c_{t}}_{G}$ represent the spring stiffness of the single flexure hinges as per Figure 1. The deflection s of the shuttle leads to differing angles $\varphi_{1}$ and $\varphi_{2}$ for the guiding and the transmission mechanism, respectively according to Equation (8). With the small angle approximation, the angle $\varphi_{1}$ can be expressed using the angle $\varphi_{2}$ (Equation (9))

(8) $$s=\tan\varphi_{1}\times l_{AD}=\tan\varphi_{2}\times l_{FG}$$

(9) $$\varphi_{1}=\varphi_{2}\frac{l_{FG}}{l_{AD}}$$

The force equation can be calculated by the first partial derivative of the elastic potential energy of the system according to the deflection angle. With the second partial derivative, the spring stiffness of all torsion springs in the entire mechanism can be calculated by:

(10) $$\frac{\partial^{2}U_{el}}{\partial \varphi_{2}{}^{2}}=({c_{t}}_{A}+{c_{t}}_{B}+{c_{t}}_{C}+{c_{t}}_{D})\frac{l_{FG}{}^{2}}{l_{AD}{}^{2}}+{c_{t}}_{F}+{c_{t}}_{G}={c_{t}}_{ges}$$

To derive the overall system stiffness, a conversion according to:

(11) $$c_{ges}=\frac{{c_{t}}_{ges}}{l_{FG}{}^{2}}$$

is required. The stiffness of the entire weighing cell can then be calculated using the following Equation (12):

(12) $$c_{ges}=\frac{1}{l_{FG}{}^{2}}(({c_{t}}_{A}+{c_{t}}_{B}+{c_{t}}_{C}+{c_{t}}_{D})\frac{l_{FG}{}^{2}}{l_{AD}{}^{2}}+c_{F}+c_{G})$$

#### 2.1.2. Numerical Stiffness Calculation

Finite element method (FEM) simulations are state of the art for the numerical calculation of complex geometries and were therefore used for comparison of the analytical calculation and further investigations. For the numerical stiffness calculation, the mechanical Ansys Parametric Design Language solver of the software ANSYS was used for a static structural analysis.

**Figure 4:**
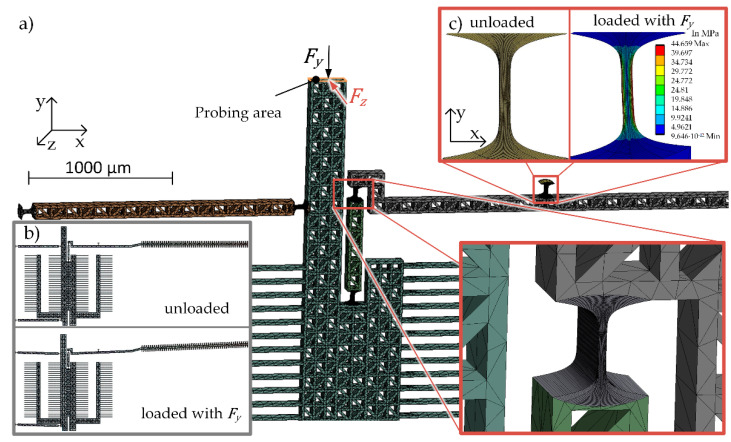
(**a**) Illustration of sections of the meshed FEM model (software ANSYS); (**b**) weighing cell in an unloaded and a loaded state with the force *F_y_*; (**c**) FEM simulation of an unloaded hinge G and the principal stress distribution if the hinge is loaded with a force *F_y_*.

For the simulation, the flexure hinges were modeled as separate solid bodies in the computer-aided design program (Autodesk Inventor) and then connected with surface-surface-contacts in ANSYS (Figure 4a). The element size of the mesh for the flexure hinges are around 18 µm^3^ and in the rigid parts around 3400 µm^3^. Parameter studies showed that these meshing combinations yield a convincing compromise between computational time and model accuracy. As model material, anisotropic silicon (density ρ = 2330 kg/m^3^, stress tensor: σ_11_ = σ_22_ = σ_33_ = 1.66 × 10^5^ MPa, τ_21_ = τ_31_ = τ_32_ = 64,000 MPa) was used directly from the ANSYS material library. The parts connected to the frame are secured with fixed supports and the force is introduced on the upper part of the shuttle, the probing area as indicated in Figure 4a. The deflection *s* is calculated at the point of the force application. The system stiffness was derived assuming a force of $F_{y}=-200$ µN in the direction of motion and the out-of-plane stiffness assuming a force of $F_{z}=-50\;$µN in out-of-plane direction, according to Figure 4 and Equation (13):

(13) $$c=\frac{F}{s}$$

### 2.2. Microfabrication of MEMS-Based Weighing Cells

**Figure 5:**
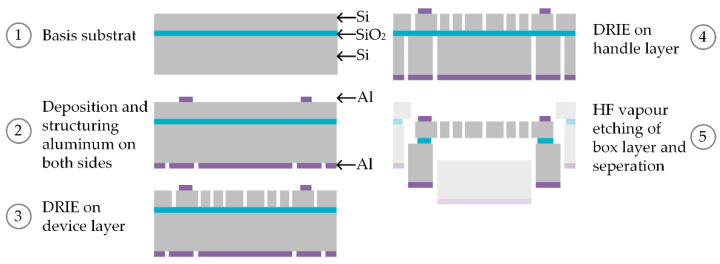
Schematic illustration of the workflow for the fabrication for a MEMS-based weighing cell based on a silicon-on-insulator substrate.

The microfabrication process of the previously described MEMS-based weighing cell is based on well-established silicon-technology and thin film processing. The workflow and the material choices for the fabrication are shown in the schematic overview in Figure 5. As basis for the fabrication process, a silicon-on-insulator (SOI) substrate was used (step 1 in Figure 5). To create an electrostatic microactuator for relatively large restoring forces and a microsensor offering a high resolution in the determination of capacitance changes, a SOI substrate with a silicon p-type device-layer (boron doped, resistivity 0.01–0.02 Ohm·cm) having a thickness of 100 µm was used. The device layer doping is required to assure a low contact-resistivity between the silicon layer and the contact pads required for signal read-out. Due to the thickness of 2 µm of the so-called box layer (silicon dioxide) and the minimized designed overlapping area of the device and the handle layer of the SOI substrate, the finally resulting friction between shuttle and handle layer can be neglected. Therefore, the handle layer represents a stabile frame with a thickness of 350 µm.

For the realization of the contact pads on the device layer, an aluminum layer of 800 nm is used (step 2 in Figure 5). A 200 nm aluminum layer is furthermore employed as a hard mask for the handle layer. The aluminum layers were deposited by sputtering (Ardenne CS400 ES PVD-Cluster tool) and structured by UV lithography using AZ1518 (MicroChemicals) as a photoresist and ANPE 80/5/5/10 (MicroChemicals) as an aluminum etchant.

After structuring the aluminum layers, the device layer is structured using a deep reactive ion etching (DRIE) process (Oxford ICP PlasmaPro100 Estrelas) and an approximately two micrometer thick resist mask (AZ1518, MicroChemicals) (step 3 in Figure 5). The etching process should create structures with steep 90° sidewalls in the 100 µm thick device layer, which will be discussed later (Section 3.1). Here, a three-step process is used, which includes an extra etching step in addition to the common cyclic deposition and etching steps. The first etching step employs a low frequency generator (50 W, 10 Hz) in addition to the inductively coupled plasma (1750 W) to remove the deposited plasma polymer in vertical direction for 500 ms with distinct physical etching. The second etching step uses just the inductive coupled plasma (2500 W) to etch the silicon for 1950 ms with distinct chemical. The box layer acts here as an efficient etch stop. After etching the remaining plasma polymers, the resist mask was removed by oxygen plasma ashing. To additionally remove the resist mask that is present under the clamped parts of the wafer, the Oxygen Plasma Cleaner 200 tool from TePla was used (20 min, 400 W).

Subsequently, the handle layer is etched by DRIE similarly to the aforementioned DRIE process but the time of the second etching step was increased to 2000 ms (step 4 Figure 5). Due to the required etch depth of 350 µm, the sidewall angle decreases. After etching, an oxygen plasma cleaning step is required on both sides of the substrate to remove remaining plasma polymers. Finally, hydrofluoric (HF) vapor is used to remove the box layer within 15 h of etching to release the movable structures of the device layer and to separate the chips from the wafer compound (step 5 in Figure 5). The working principal of the separation process is adapted from [19]. A 40 wt% HF acid was used for this purpose at 50 °C to etch the silicon dioxide uniformly.

### 2.3. Experimental Determination of the System Stiffness

**Figure 6:**
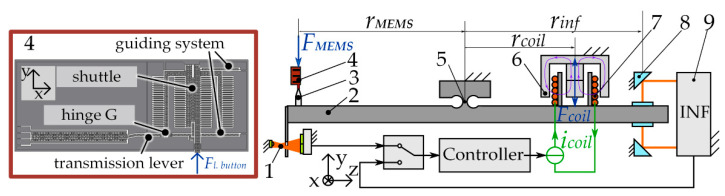
Illustration of the direct force measurement device used for measuring the stiffness of the MEMS-based weighing cell: (1) slit aperture; (2) beam balance; (3) loading button; (4) MEMS-based weighing cell chip; (5) joint; (6) permanent magnet; (7) coil; (8) deflection mirror; (9) interferometer.

The smallest uncertainty in measuring the spring stiffness in microsystems is given by the static method of direct force measurement and is in the range of 0.5 to 4%. A device that implements this method for cantilever stiffness measurement was developed and studied at TU Ilmenau as published elsewhere [20,21,22]. The schematic illustration of the measuring device is shown in Figure 6.

By replacing the original cantilever holder of the device with a specially designed holder for the MEMS-based weighing cell, force-displacement measurements were realized and used to determine its stiffness.

When the MEMS based guiding mechanism (4) acts on the loading button (3), the aluminum-based balance (2) deflects from its zero position. This deflection is detected by means of a slit aperture (1) and a differential interferometer (9) (from SIOS Messtechnik). The deflection signal in the form of an analog voltage or a digital quadrature signal is transmitted to the controller that compensates the deviation from the set position. This is done by transferring an according compensation voltage to a voltage-current converter, which is connected to the coil (7) in a constant magnetic field (6). Due to the occurrence of Lorentz forces, the balance returns to its zero setting. The compensation current is read-out with a multimeter and finally used to determine the stiffness. The main advantage of this measuring device is the linearity of forces. The elastic force of the MEMS-based weighing cell is calculated with the following Equation:

(14) $$F_{MEMS}=\frac{B\times l_{coil}\times r_{coil}}{r_{MEMS}}i_{coil}$$

where B is the magnetic-flux density, $l_{coil}$ the length of the coil, $r_{coil}$ distance from the balance hinge to the coil and $r_{MEMS}$ the distance from the joint to the loading button.

**Figure 7:**
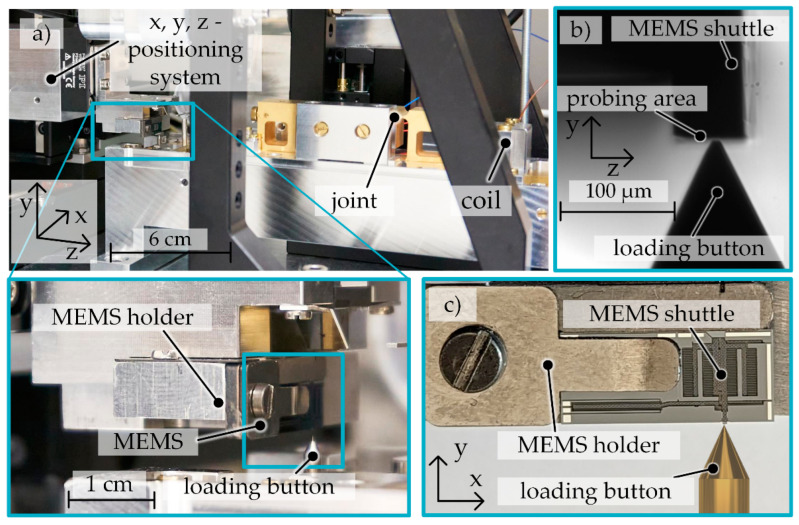
Force-displacement measurement device used for the stiffness measurement of the MEMS based weighing cell (**a**) overall with a magnification to the MEMS holder and the loading button; (**b**) side view from the camera used for positioning (**c**) front view with the MEMS.

The positioning of the MEMS-based weighing cell relative to the loading button in the directions *x*, *y*, *z* (according to Figure 7) is performed by means of three linear positioning stages (from Physik Instrumente). After adjusting the position of the MEMS-based weighing cell, its movement along the *y*-axis occurs due to the use of a piezoelectric actuation principle with a maximum movement range of 100 µm but a high positional resolution of 100 pm. To determine the stiffness of the MEMS-based weighing cell, the setup uses an additional differential interferometer (from SIOS Messtechnik) to record the displacement of the weighing balance signal. Figure 7a shows the MEMS-based weighing cell being positioned in the measurement device, Figure 7b a side view from the camera (from Thorlabs) used to position the sample and Figure 7c the front view of the MEMS based weighing cell. The MEMS holder was specially designed for the MEMS-based weighing cell and enables the stiffness measurement in direction of motion and in out-of-plane direction by alternative positioning of an integrated holding element.

## 3. Results and Discussion

### 3.1. Theoretical Stiffnesses Evaluation

To evaluate the stiffness as a function of the microsystem geometry, systems as shown in Figure 1b with three different hinge geometries and differing lever lengths were manufactured. The two systems labeled W 3-1 C1 and C2 are identical, W 3-2 and W 3-3 have different hinge parameters and W 2-1 has different lever lengths compared to the first two systems, as shown in Table 1. It is expected, that due to the longest and thinnest hinges of system W 3-2, its stiffness in direction of motion must be the smallest in comparison to the other systems. The hinges of W 3-3 are shorter and much thicker, which should lead to the highest system stiffness in direction of motion. These assumptions are consistent with the analytical and numerical results as presented in Table 1.

The stiffness of the single hinges obtained with the detasFlex software, which were used for the analytical calculation are shown in Table 1. Due to the inherent mechanical stops, which limit the movement of the shuttle to maximum 5 µm, the maximum deflection of the hinges is 0.2°. Hinge G undergoes the largest deflection of the entire MEMS-based weighing cell mechanism and was therefore investigated further. For example, a maximum strain of 0.041% in hinge G of W 2-1 was obtained with the detasFlex software. In the numerical solution the hinges are analyzed regarding the stress of 44 MPa (see Figure 4c) which leads to a strain of 0.044%. Both results are considerably lower than the maximum allowable strain of 0.27% for silicon (as specified within the detasFlex software).

The analytical and numerical results differ with a maximum of 3.8%. The stiffness ratio is a means of describing the relation of the stiffness in out-of-plane motion to the stiffness in the direction of motion. As the stiffness of the individual hinges increases in the direction of motion, the stiffness in the out-of-plane direction also increases, but not to the same degree. Depending on the hinge parameters the stiffness ratio varies from 1.8 for the stiffest system to 10.8 for the system with the lowest stiffness in direction of motion.

### 3.2. Analysis of the Influences of Fabrication Tolerances on the System Stiffness

**Figure 8:**
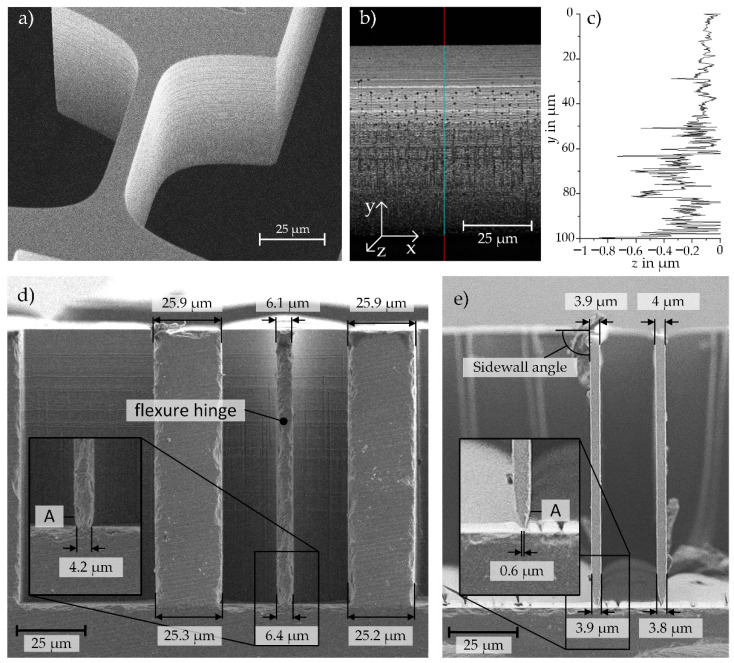
(**a**) scanning electron microscopy (SEM) images showing a flexure hinge, (**b**) exemplary device layer sidewall image as recorded by laser-scanning microcopy (LSM), and (**c**) an according topography profile, (**d**) SEM image of the cross section of a cutted flexure hinge with a designed height of *h* = 7 µm, (**e**) SEM image of the cross section with a designed height of *h* = 5 µm.

As discussed before, geometry deviations have a significant influence on the stiffness of the weighing cell. Therefore, the lateral and vertical geometry deviations of the fabrication process are analyzed in detail in the following section and an overview of the measured geometries are shown in Figure 8.

#### 3.2.1. Measured Influencing Geometry Parameters

The lateral geometry is around 0.5 to 1 µm smaller than specified in the design (Figure 8a,d,e). A possible reason for these differences is the microfabrication process. The structures are fabricated by hard contact UV lithography, where the gap between the mask and the substrate, due to its pre-patterning, yields lateral geometry deviations. A small deviation of about 0.5 µm can already be seen in the resist mask before etching. Furthermore, a very small under-etching of the mask was observed after the DRIE process. This effect was reduced primarily by adjusting the first five etch loops in the direction of more passivation and less etching.

Vertical geometry deviations resulting from the DRIE process are exemplarily shown in Figure 8d,e). A sidewall angle of 89.5 to 90.3 degrees is measured within the resolution of the scanning electrode microscope (SEM). For the length measurements, the thickness of the device layer with 100 µm was used as reference length. A reduction in the hinge height of up to 3 µm, due to a slight over-etching during the DRIE process, can be seen on the last 2–3 µm of the hinge structures as marked with the letter A in Figure 8d,e. Riffles, which are a result of the DRIE process, can also have an influence on the stiffness of the system and were evaluated through the roughness of the sidewall. The profile of the sidewalls was recorded using a 3D laser scanning microscope (LEXT 4100, Olympus, laser spot size of 200 nm) and the roughness was accordingly analyzed (Figure 8b,c). For the upper 50 µm of the hinge width, the roughness is around a quarter (*R_z_* = 0.14 µm) of the roughness of the lower 50 µm etch depth (*R_z_* = 0.57 µm). Since the etch rate decreases in depth with longer etching and ends at 200 cycles for the 100 µm thick component layer, the etch riffles got smaller the deeper the structures are etched.

#### 3.2.2. Sidewall Angle Influence on the Stiffness

Due to the manufacturing process of deep reactive ion etching (DRIE), see Section 2.3, it is likely that the sidewall angle of the etched feature, e.g., the comb-capacitor plates and hinges, are smaller than 90° [23]. The sidewall angel represents a consistent height h of the flexure hinge over the whole width.

To evaluate the influence of a sidewall angle, as marked in Figure 8e, being smaller than 90° on the stiffness of the entire MEMS-based weighing cell, the stiffness of single flexure hinges with different sidewall angles and hinge heights h was investigated by means of FEM simulations. The force application point is 200 µm away from the rotating center of a flexure hinge and, thus, the stiffness as per Equation (13) needs to be converted using Equation (11).

**Figure 9:**
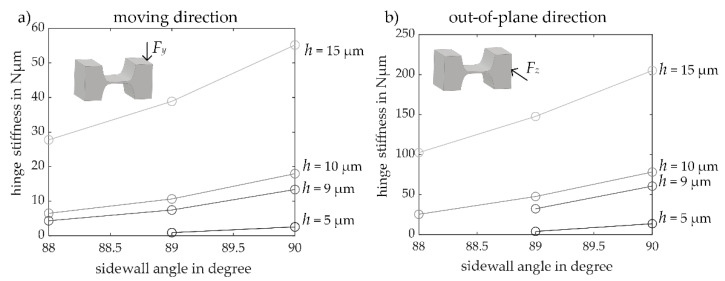
Dependence of the etching quality on the single flexure hinge stiffness, (**a**) in direction of motion and (**b**) in out-of-plane direction.

Comparing the numerical stiffness in direction of motion of a single flexure hinge with a sidewall angle of 90 ° determined by means of FEM simulation with the calculation results based on the detasFLEX software, deviations of −2.3 to 1.1% are obtained. The studies have also shown that the stiffness decreases with decreasing sidewall angle as shown in Figure 9.

Furthermore, it was observed that the smaller the height of the flexure hinge, the larger are the deviations resulting from different sidewall angles in a range of just one degree. For instance, for a hinge height of 15 µm the influence of a deviation of one degree in sidewall angle results in a deviation of 30% for the stiffness. For a hinge height of 5 µm, the deviations are already around 65%. Regarding the out-of-plane motion direction, the same behavior can be observed for the correlation between stiffness and etching angle (Figure 9b). Additionally, it was observed that the unintentional out-of-plane movement of the shuttle during its desired in-plane motion increases as the sidewall angles become smaller. These results show clearly, that a distinct focus must be set during the microfabrication process on controlling the steep sidewall angle, as mentioned before in Section 2.3.

### 3.3. Experimental Investigation of the System Stiffness

The stiffness of the MEMS-based weighing cell is measured using the previously described force-displacement measuring device (Figure 6 and Figure 7a). The weighing cell was loaded in several cycles by means of the loading button within five steps of 0.4 µm each to reach an overall travel range of 2 µm. The unloading was released in the same manner, with each step again being held for 30 s. The displacement and corresponding compensating current are measured throughout the entire experiment. Additionally, to verify the long-term stability of the stiffness of the MEMS-based weighing cell, a long-term measurement was performed for 9.5 h.

**Figure 10:**
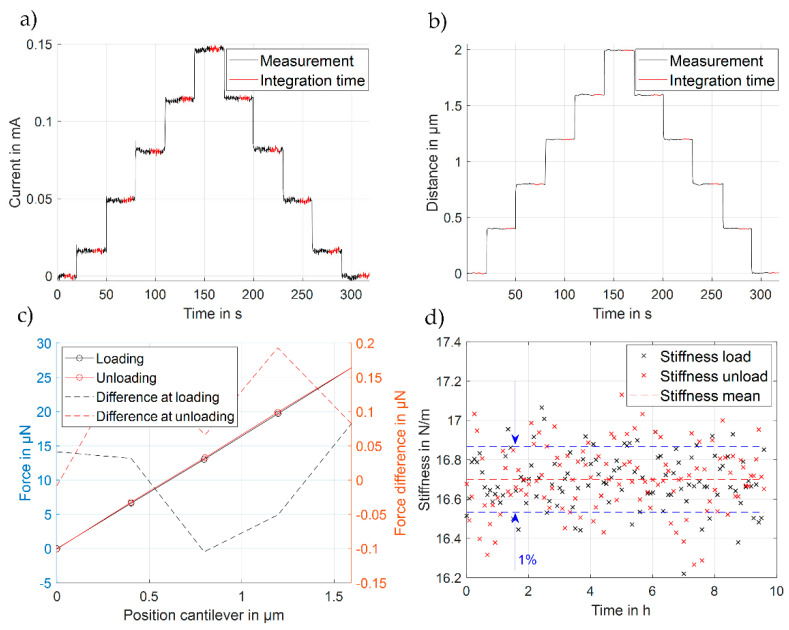
(**a**) Recorded compensation current signal from one cycle with 10 s integration time; (**b**) corresponding measured displacement signal MEMS of one calibration cycle with 10 s integration time; (**c**) Force-displacement characteristics per cycle; (**d**) long-term measurement of MEMS stiffness of the system W 3-1 C1.

The movement of the shuttle of the MEMS-based weighing cell and the compensating current of one of the cycles during the long-term measurement of system W 3-1 C1 are shown in Figure 10a,b. The compensation current in the first step differs from the other steps as shown in Figure 10a, which is because the MEMS-based weighing cell is not initially in contact with the loading button. To avoid impacts from cohesive forces on the measurement results, the first step of data processing is therefore not considered in the linear regression-based analysis. Respective measurements of the force-displacement characteristic for one cycle and for the stiffness values during the long-term measurement are shown in Figure 10c,d.

**Figure 11:**
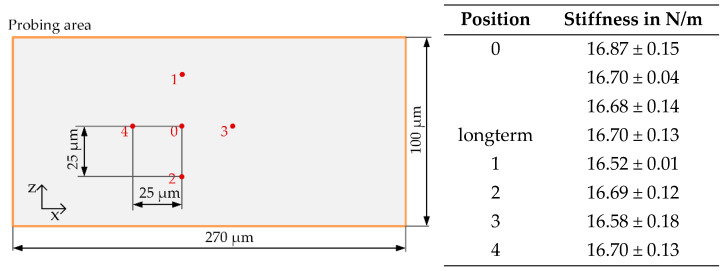
Eccentric test, (**left**): geometrical illustration of probing area, (**right**): stiffness measurement results in direction of motion of system W 3-1 C1.

The difference between the loading and unloading force is measured to be approximately 25 µN per cycle (Figure 10c). A slight hysteresis occurred during the measurement in loading and unloading direction, mainly from the force displacement device itself due to creeping effects of the aluminum alloy (AlZnMgCu1.5) balance but not from the MEMS-based weighing cell. The long-term measurement of the MEMS stiffness lasted in total for 150 cycles (9.5 h) without noticeable drift, indicating the overall stability of the sample and of the measurements itself (Table in Figure 11). The measurement yielded finally a value of 16.70 ± 0.13 N/m.

The guiding system of the MEMS-based weighing cell mechanism has been designed in such a way that the force measurement is invariant to the force application point on the probing area. Accordingly, the stiffness measurement of the MEMS-based weighing cell should be independent of the force application point as well. To verify this assumption a so-called eccentric test was conducted. For this, four additional force application points are used for measuring the stiffness, as shown in Figure 11. The zero point “0” was set in z-direction with an accuracy of ± 2 µm (cf. Figure 7b). The positioning in *x*-direction could not be done with the same precision based on the setup design, which was originally built for measurements in one direction only. The results of the eccentric test of system W 3-1 is exemplarily shown in Figure 11. The deviations of the stiffness measurements in position “0” are 0.77% and the deviations of the other points are in the range of 1.67%. This supports the hypothesis that the stiffness measurement is invariant to the position of the force application point. Accordingly, only the results of the stiffness measured in point “0” are used for further measurements.

### 3.4. Discussion of the Theoretical and Experimental Results

Based on the previous measurements done in the Section 3.2.1 and Section 3.2.2, certain parameters were adapted in the theoretical calculation to allow appropriate comparison with the experimental results.

The measurements showed that the fabricated flexure hinges have slightly lower hinge heights than specified in the original design, cf. Figure 8. Therefore, a reduction of the hinge heights of 0.5 µm was assumed for the stiffness calculations. The sidewall angle has also a non-negligible influence on the stiffness of the systems, cf. Figure 9. In the previous analysis of the fabricated test specimen, sidewall angles between 89.5° and 90.3° were measured. These values vary around the ideal sidewall angle of 90° and therefore no adjustment is made here for the calculation of the stiffness. In summary, this means that for further calculations the lateral geometry was adjusted but the vertical ones are not.

**Figure 12:**
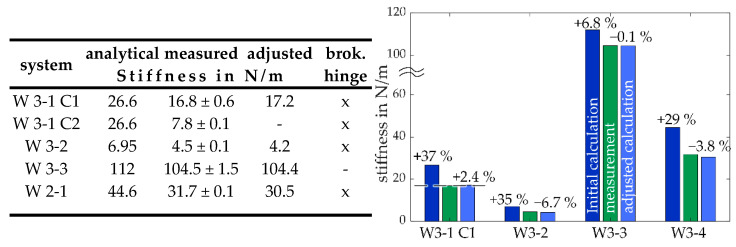
Stiffness measured in the direction of motion compared to the calculated analytical values. (**Left**): values of the stiffness results, (**right**): percentage representation of the deviation from the measured stiffness (green) to the analytical stiffness with ideal geometry (dark blue) and to the calculations using the adjusted geometry (light blue).

The deviations between the calculated stiffnesses of the MEMS-based weighing cells and the experimentally determined stiffnesses range from 6.8 to 70%, (Figure 12).

During the stiffness measurements, it is not possible to observe the movement of the shuttle of the MEMS-based weighing cell. Accordingly, any system deterioration, such as breakage of a hinge or even of the entire system, cannot be monitored and thus not prevented. Subsequent inspections of the systems have shown a damaged flexure hinge A W3-1 and W2-1 and a complete breakage of the systems W 3-1 C2 and W 3-2. The later occurred during the out-of-plane stiffness measurement. Therefore, the following discussion is partly based on theoretical assumptions.

System W 3-1 C2 and W 3-1 C1 are expected to have the same stiffness due to their identical geometry. However, this could not be shown in the measurements (Figure 12), and due to the complete fracture of the system W 3-1 C2 during the out-of-plane measurement, no further investigations regarding the distinct difference in the stiffness can be made. It is likely, that the system was already partially broken while performing the stiffness measurement in the direction of motion. The measured stiffness of the systems W 3-1 C1, W 3-2, and W 2-1 show deviations from the calculated stiffness of 29% to 37%. Due to the broken hinges in the lower lever of the systems W 3-1 C1 and W 2-1 and assuming the same of W 3-2, the Equation (12) can be adjusted accordingly for the stiffness calculation, removing the stiffness $c_{A}$ and $c_{D}$. The deviation between measurement and adjusted stiffness calculation is more suitable, which yields values between −6.7% and 3.8%. For system W 3-3, for which no breakage was detected and which is the most robust system due to a hinge height of *h* = 15 µm, sufficient agreement between the calculated and measured stiffness was demonstrated with a deviation of only 0.1%. 

## 4. Summary and Conclusions

The design, fabrication and mechanical analysis of functional MEMS-based weighing cells was demonstrated. For microfabrication, silicon-on-insulator substrates and deep reactive ion etching (DRIE) with optimized process parameters were used to fabricate, for example, the required comb capacitors, shuttle and bending hinges. The system was particularly studied in terms of in-plane and out-of-plane stiffness, which is a crucial parameter. A rigid body model for the stiffness calculation was presented and discussed to efficiently determine the stiffness of the entire microsystem and for a reduced MEMS-based weighing cell mechanism. Numerical simulations based on the finite element method were performed for comparison purposes. The system stiffness analysis was performed taking into account the manufacturing deviations. The analytical results were in convincing agreement with the simulations, with deviations ranging from 0.04 to 3.8%. The microfabricated MEMS-based weighing cells were furthermore also experimentally characterized with respect to in-plane and out-of-plane stiffness using a force-displacement measurement approach. The measured stiffness in the direction of motion showed with deviations in the range of 0.1 to 6.7% sufficient agreement with the analytical results.

However, an improved experimental characterization approach is needed to avoid breakage of MEMS components and to improve the overall robustness of the measurement. Nevertheless, we demonstrated that MEMS-based weighing cells can be successfully realized in analogy to their macroscopic counterparts, which provides a solid basis for further research in this field.

## Figures and Tables

**Table 1:** Geometrical parameters according to Figure 2 and Figure 3 (*w* = 100 µm) and theoretical stiffness results for a single hinge and for the entire MEMS-based weighing cell systems in direction (dir.) of motion (−y) and out-of-plane direction (−z), according to Figure 4a).

System	Hinge Size in µm	Lever Size in µm	Dir.	Stiff. Single Hinge in Nµm	Analytical Solution in N/m	Numerical Solution in N/m	Deviation in%	Stiffness Ratio
W 3-1 C1/C2	*h* = 9 *l* = 100	*l_AD_* = 2000 *l_FG_* = 1400	−y	13.18	26.6	27.6	3.8	5.1
			−z		-	139.7	-	
W 3-2	*h* = 7 *l* = 200	*l_AD_* = 2000 *l_FG_* = 1400	−y	3.27	6.95	7.2	3.6	10.8
			−z		-	78	-	
W 3-3	*h* = 15 *l* = 100	*l_AD_* = 2000 *l_FG_* = 1400	−y	55.46	112	111.95	0.04	1.8
			−z		-	200.4	-	
W 2-1	*h* = 9 *l* = 100	*l_AD_* = 1700 *l_FG_* = 1000	−y	13.18	44.6	45.5	2.0	4.2
			−z		-	192.3	-	

## Data Availability

Not applicable.
